# Low educational attainment is associated with higher all-cause and cardiovascular mortality in the United States adult population

**DOI:** 10.1186/s12889-023-15621-y

**Published:** 2023-05-16

**Authors:** Najah Khan, Zulqarnain Javed, Isaac Acquah, Kobina Hagan, Madiha Khan, Javier Valero-Elizondo, Ryan Chang, Umair Javed, Mohamad B. Taha, Michael J. Blaha, Salim S. Virani, Garima Sharma, Ron Blankstein, Martha Gulati, Elias Mossialos, Adnan A. Hyder, Miguel Cainzos Achirica, Khurram Nasir

**Affiliations:** 1 Department of Internal Medicine, Houston Methodist Hospital, Houston, TX) USA; 2 Center for Cardiovascular Computational Health and Precision Medicine (C3-PH) , Houston Methodist, Houston, TX) USA; 3 Houston Methodist Academic Institute, Houston Methodist, Houston, TX) USA; 4 Division of Cardiovascular Prevention and Wellness, Houston Methodist DeBakey Heart and Vascular Center, 6550 Fannin St Suite 1801, Houston, TX 77030 USA; 5 Department of Medicine, MedStar Union Memorial Hospital, Baltimore, MD 21218 USA; 6 Washington University in St. Louis, St. Louis, MO) USA; 7 National University of Medical Sciences, Rawalpindi, Pakistan; 8 Ciccarone Center for the Prevention of Cardiovascular Disease, Johns Hopkins University School of Medicine, Baltimore, MD) USA; 9 Michael E. DeBakey Veterans Affairs Medical Center and Baylor College of Medicine, Houston, TX) USA; 10 Cardiovascular Imaging Program, Department of Medicine and Radiology, Brigham and Women’s Hospital, Boston, MA) USA; 11 Department of Preventive Cardiology, Harbor-UCLA Medical Center, Los Angeles, CA USA; 12 Department of Health Policy, London School of Economics and Political Science, London, UK; 13 Center On Commercial Determinants of Health, Milken Institute School of Public Health, The George Washington University, Washington, DC USA; 14 Department of Cardiology, Hospital del Mar / Parc de Salut Mar, Barcelona, Spain

**Keywords:** All-cause mortality, Cardiovascular disease, Educational attainment, Health disparities

## Abstract

**Introduction:**

Educational attainment is an important social determinant of health (SDOH) for cardiovascular disease (CVD). However, the association between educational attainment and all-cause and CVD mortality has not been longitudinally evaluated on a population-level in the US, especially in individuals with atherosclerotic cardiovascular disease (ASCVD). In this nationally representative study, we assessed the association between educational attainment and the risk of all-cause and cardiovascular (CVD) mortality in the general adult population and in adults with ASCVD in the US.

**Methods:**

We used data from the 2006–2014 National Death Index-linked National Health Interview Survey for adults ≥ 18 years. We generated age-adjusted mortality rates (AAMR) by levels of educational attainment (< high school (HS), HS/General Education Development (GED), some college, and ≥ College) in the overall population and in adults with ASCVD. Cox proportional hazards models were used to examine the multivariable-adjusted associations between educational attainment and all-cause and CVD mortality.

**Results:**

The sample comprised 210,853 participants (mean age 46.3), representing ~ 189 million adults annually, of which 8% had ASCVD. Overall, 14.7%, 27%, 20.3%, and 38% of the population had educational attainment < HS, HS/GED, Some College, and ≥ College, respectively. During a median follow-up of 4.5 years, all-cause age-adjusted mortality rates were 400.6 vs. 208.6 and 1446.7 vs. 984.0 for the total and ASCVD populations for < HS vs ≥ College education, respectively. CVD age adjusted mortality rates were 82.1 vs. 38.7 and 456.4 vs 279.5 for the total and ASCVD populations for < HS vs ≥ College education, respectively. In models adjusting for demographics and SDOH, < HS (reference =  ≥ College) was associated with 40–50% increased risk of mortality in the total population and 20–40% increased risk of mortality in the ASCVD population, for both all-cause and CVD mortality. Further adjustment for traditional risk factors attenuated the associations but remained statistically significant for < HS in the overall population. Similar trends were seen across sociodemographic subgroups including age, sex, race/ethnicity, income, and insurance status.

**Conclusions:**

Lower educational attainment is independently associated with increased risk of all-cause and CVD mortality in both the total and ASCVD populations, with the highest risk observed for individuals with < HS education. Future efforts to understand persistent disparities in CVD and all-cause mortality should pay close attention to the role of education, and include educational attainment as an independent predictor in mortality risk prediction algorithms.

**Supplementary Information:**

The online version contains supplementary material available at 10.1186/s12889-023-15621-y.

## Introduction

Atherosclerotic cardiovascular disease (ASCVD) is the leading cause of death in the US [1]. While traditional risk factors for ASCVD have been extensively researched [2], socioeconomic and contextual factors which shape cardiovascular health – directly and indirectly – are less well studied [3]. Collectively, these factors are known as social determinants of health (SDOH) [3, 4]. Unfavorable SDOH have been shown to increase the risk of poor health outcomes – including CVD – with recent evidence suggesting that social disadvantage may increase the risk of all-cause mortality, independent of demographics or clinical risk factors [5].

Educational attainment is an important SDOH and a reliable measure of socioeconomic status. Lower educational attainment has a well-established association with higher risk of ASCVD [6–9] and is inversely associated with all-cause and cardiovascular disease (CVD) mortality, with higher educational attainment associated with lower mortality rates [10–15]. The magnitude of lower educational attainment or less than high school education has been associated with higher all-cause and cardiovascular mortality in several studies [16].

However, relatively little is known about the longitudinal impact of educational attainment on all-cause and CVD mortality specifically in the United States, particularly at the population level and using most contemporary data. Also, when evaluating the education-mortality association, prior studies typically performed a partial adjustment for confounders and sociodemographic factors and only a few assessed the potential mediation role of CVD risk factors, thereby preventing a full appreciation of the impact education has on mortality [5, 10–15]. Finally, few studies have evaluated these associations specifically among individuals with ASCVD [10–12], which remains the number one cause of death in the US.

Our aim was to conduct a large-scale, nationally representative study assessing the association between varying levels of educational attainment and all-cause and CVD mortality in the general US adult population, and specifically among individuals with ASCVD, over a 10-year period. We also aimed to assess these associations specifically across relevant sociodemographic subgroups.

## Methods

### Research design and study population

Our study included individuals ≥ 18 years of age from the 2006–2014 National Health Interview Survey (NHIS), an annual multistage survey conducted in the civilian non-institutionalized population of the United States. Details on survey design and information is available from https://www.cdc.gov/nchs/nhis/1997-2018.htm [17]. Data for adults who completed the surveys between 2006–2014 was linked to the National Death Index (NDI) through December 2015. The NDI is a centralized database of US death record information and includes detailed data on underlying cause of death. The following identifiers were used to link NDI to NHIS: social security number, first name, last name, father’s surname, month/day/year of birth, sex, race, state/country of birth, and residence [17]. We excluded individuals with missing data on NDI linkage (ineligible for NDI linkage), education, income, and other/unidentified racial/ethnic category.

### Research ethics

This research does not contain Human Subjects. Due to the de-identified nature and public availability of NHIS data, this study was exempt from review by the institutional review board of Houston Methodist. No administrative approval was needed to access the raw data as it is publicly available. All data was anonymized prior to use in this study.

### Study exposure

Our exposure variable, self-reported educational attainment, was categorized as a 4-level ordinal variable: (1) no high school diploma (< HS), (2) HS diploma or general education development equivalent (HS/GED), (3) some college, and (4) at least one college degree (≥ college).

### Ascertainment of baseline ASCVD

Respondents with an affirmative answer to at least one of the following items were deemed to have ASCVD: “Have you ever been told by a doctor or other health professional that you had coronary heart disease…angina or angina pectoris…a heart attack (also called a myocardial infarction) …a stroke?”. This information was used to define the ASCVD subpopulation as a binary “yes/no” variable.

### Outcomes

Two outcomes of interest were evaluated: all-cause and CVD mortality. For decedents, mortality type (all-cause and CVD morality) was ascertained using death certificate records of the International Classification of Disease (ICD) codes for the primary cause of death. All-cause mortality was death due to any cause. CVD mortality was defined as death with CVD (I00-I09, I11, I13, I20-I51) as the primary cause of death.

### Other relevant variables

Other study variables used in our analyses included age categories (18–39, 40–64, ≥ 65 years) [18, 19], sex (men, women), race/ethnicity (non-Hispanic White [NHW], non-Hispanic Black [NHB], and Hispanic), cardiovascular risk factors, family income (high, middle, low, and lowest income) and insurance type (uninsured, private insurance, or public insurance) these were included as covariates. Cardiovascular risk factors included hypertension, diabetes, obesity (body mass index ≥ 30 kg/m^2^), current smoking status, and insufficient physical activity. Insufficient physical activity included any of: < 150 min per week of moderate-intensity aerobic physical activity; < 75 min per week of vigorous-intensity aerobic physical activity, or < 150 min per week of moderate to vigorous-intensity aerobic physical activity. Family income was categorized based on the percentage of family income relative to the federal poverty limit from the US Census Bureau, i.e., high income (≥ 400%) of FPL, middle income (200%– < 400%), low income (125%– < 200%), and lowest income (< 125%) of FPL.

### Statistical analysis

We reported the descriptive characteristics of the sample by educational attainment and compared the differences in these statistics with chi-squared tests. We generated weighted proportions of participant characteristics by levels of educational attainment to obtain nationally representative estimates.

We reported age-adjusted mortality rates (AAMR) per 100,000 person-years with 95% confidence intervals, by levels of educational attainment, using Poisson regression models. We examined two outcomes: all-cause and CVD mortality. First, we reported AAMRs for each mortality outcome in the total population. Second, we reported AAMRs in adults with ASCVD.

Kaplan–Meier survival curves were used to plot survival by levels of educational attainment during the 10 years of follow-up. Multivariable Cox proportional hazards models were used to examine the association between educational attainment and all-cause and CVD mortality separately in the total population and in adults with ASCVD. Four models were tested: Model 1: unadjusted; Model 2: adjusted for age, sex and race/ethnicity; Model 3: adjusted for Model 2 + insurance and income; Model 4: adjusted for Model 3 + traditional cardiovascular risk factors (hypertension, diabetes, obesity (body mass index ≥ 30 kg/m2), current smoking status, and insufficient physical activity).

## Results

Our final sample consisted of 210,853 adults, representing 189 million annualized US adults. Descriptive characteristics of the study population are summarized in Table 1. Overall, 14.7%, 27%, 20.3%, and 38% of the population had < HS, HS/GED, some college, and ≥ college respectively. Participants with ≥ college degree were more likely to be female, non-Hispanic White, and have a lower burden of CVD risk factors than those with < HS education. Conversely, participants with < HS education were more likely to belong to families with low income and have no health insurance coverage.

**Table 1:** Descriptive characteristics of adults from the National Health Interview Survey, 2006–2014

	< High School	High School or GED	Some College	≥ College	*p*-value
Sample (N)	35,678	55,952	42,175	77,048	
Weighted sample, (weighted %)	27,827,551 (14.7)	51,029,685 (27.0)	38,263,821 (20.3)	71,701,173 (38.0)	
Age Category, n (weighted %)					< 0.001
18–39	12,129 (38.8)	19,173 (37.3)	19,160 (49.0)	28,375 (37.2)	
40–64	13,389 (37.8)	24,339 (44.5)	16,471 (38.7)	36,711 (49.7)	
65 & Above	10,160 (23.4)	12,440 (18.2)	6,544 (12.4)	11,962 (13.1)	
Sex, n (weighted %)					< 0.001
Male	16,257 (51.0)	25,801 (50.2)	18,131 (46.5)	34,276 (48.1)	
Female	19,421 (49.0)	30,151 (49.8)	24,044 (53.5)	42,772 (51.9)	
Race/Ethnicity, n (weighted %)					< 0.001
Non-Hispanic White	13,418 (48.0)	35,626 (71.5)	28,017 (73.5)	59,045 (82.2)	
Non-Hispanic Black	6,798 (14.6)	10,226 (13.9)	7,885 (14.3)	9,935 (9.7)	
Hispanic	15,462 (37.4)	10,100 (14.6)	6,273 (12.2)	8,068 (8.1)	
Cardiovascular risk factors, n (weighted %)					
Hypertension	14,024 (35.2)	20,045 (32.7)	12,521 (26.8)	20,983 (25.8)	< 0.001
Diabetes	5,535 (14.0)	6,159 (10.0)	3,640 (7.7)	5,394 (6.4)	< 0.001
Smoker	8,783 (26.9)	14,667 (27.1)	9,204 (21.3)	9,444 (11.7)	< 0.001
Obesity	12,719 (34.9)	19,539 (34.6)	13,896 (32.0)	21,461 (27.0)	< 0.001
Insuff. Active	25,542 (70.8)	34,535 (61.6)	21,144 (50.0)	31,113 (40.2)	< 0.001
Family Income, n (weighted %)					< 0.001
Lowest income	17,597 (41.2)	14,498 (20.1)	10,052 (18.2)	7,153 (6.7)	
Low-income	7,977 (23.3)	10,564 (17.7)	6,485 (13.7)	6,562 (7.2)	
Middle-income	7,589 (25.4)	18,827 (35.8)	13,655 (33.6)	21,097 (25.9)	
High-income	2,515 (10.2)	12,063 (26.4)	11,983 (34.4)	42,236 (60.2)	
Insurance, n (weighted %)					< 0.001
Uninsured	10,943 (32.7)	11,712 (21.8)	7,150 (16.8)	7,090 (8.5)	
Private	6,541 (23.9)	22,603 (47.6)	22,332 (61.5)	52,558 (75.0)	
Medicare	8,476 (21.9)	12,588 (19.7)	6,863 (13.9)	11,987 (13.6)	
Medicaid	8,505 (21.0)	6,933 (10.6)	3,776 (7.5)	2,705 (2.7)	
Other	131 (0.5)	180 (0.3)	108 (0.3)	89 (0.1)	

*Abbreviations*: *ASCVD* Atherosclerotic cardiovascular disease, *CVD* cardiovascular disease, *GED* General Equivalency Diploma, *HS* high school, *Insuff* Insufficiently

### Age-Adjusted Mortality Rates

**Fig. 1:**
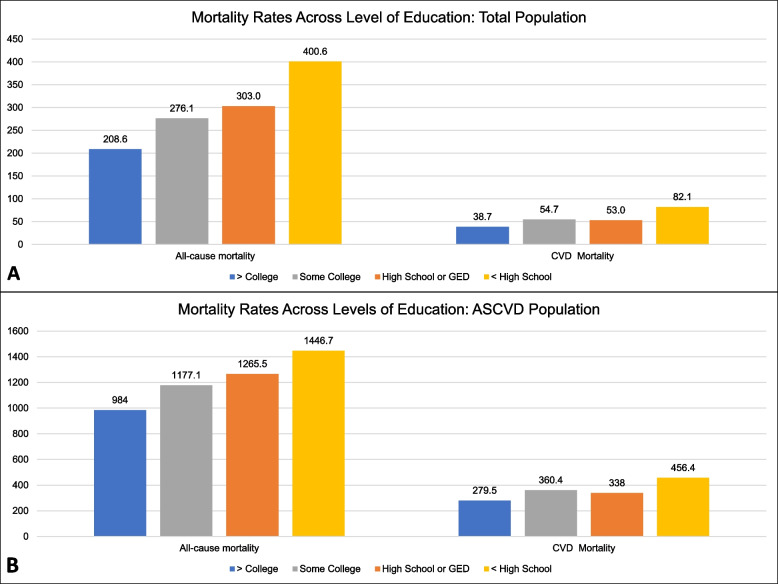
Age-adjusted mortality rates per 100,000 person-years across levels of education: Total Population (**A**) and ASCVD Population (**B**). ASCVD, Atherosclerotic cardiovascular disease; CVD, cardiovascular disease; GED, General Equivalency Diploma; HS, high school

AAMRs for all-cause and CVD mortality by educational attainment after a median follow-up duration of 4.5 years (IQR: 2.5–6.4) are presented in Fig. 1 (panels A and B) and Supplementary Table S3, Additional file 1. AAMRs increased with decreasing educational attainment in the total population (Fig. 1A) and the ASCVD population (Fig. 1B). All-cause and CVD AAMRs were approximately twofold higher for < HS vs. ≥ College in the total population and 1.5 to twofold higher in the ASCVD population. AAMRs for < HS vs. ≥ College in the total population were 400 vs. 209 and 82 vs. 39 per 100,000 person-years (PYs) for all-cause and CVD mortality, respectively. All-cause and CVD AAMRs for < HS vs. ≥ College were 1447 vs. 984 and 456 vs. 280 per 100,000 PY in the ASCVD population. The observed education-mortality patterns (i.e., increasing mortality rates with lower education attainment) were similar among individuals in the total and ASCVD population.

**Fig. 2:**
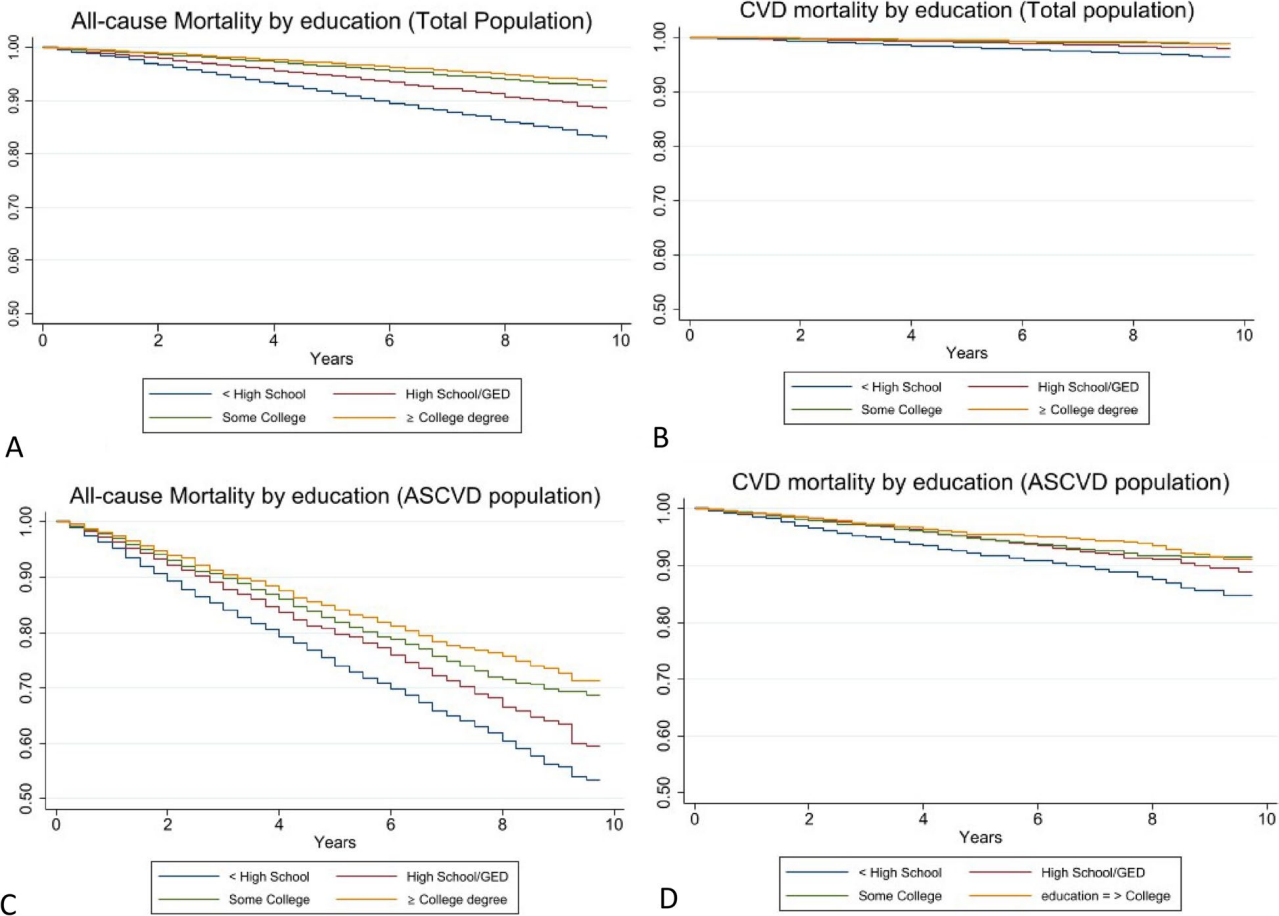
Ten-year survival rates by population and mortality. **A**-**B **Total Population and **C**-**D **ASCVD Population. ASCVD, Atherosclerotic cardiovascular disease; CVD, cardiovascular disease; GED, General Equivalency Diploma; HS, high school

Findings from the Kaplan Meier analysis are shown in Fig. 2. We found a positive association between educational attainment and 10-year survival for all population subgroups and mortality types. Ten-year survival rates for < HS vs. ≥ College were 94% vs. 83% and 99% vs. 96%, respectively for all-cause and CVD mortality in the total population. Survival rates were the lowest in the ASCVD population for all-cause mortality for < HS vs. ≥ College (53% vs 75%).

### Multivariable-adjusted analyses

Results from multivariable regression are shown in Table 2. In models adjusted for age, sex, race/ethnicity, income, and insurance status (Model 3), participants with < HS had 1.4-fold (HR = 1.39; [95% CI 1.29, 1.50]) and 1.5-fold (HR = 1.51; 95% CI 1.26, 1.81) higher risk of all-cause and CVD mortality, in the total population, relative to those with ≥ College. Similar patterns were seen for the ASCVD population. In the ASCVD population, participants with < HS had 1.2-fold (HR = 1.21; 95% CI 1.06, 1.38) and 1.4-fold (HR = 1.38; 95% CI 1.04, 1.82) higher risk of all-cause and CVD mortality relative to those with ≥ College. Important to note, the education-mortality association was only statistically significant for < HS (compared to other educational attainment levels) for all-cause and CVD mortality in the ASCVD population and CVD mortality in the total population.

**Table 2:** Hazard ratios of the association between educational attainment and risk of all-cause and cardiovascular mortality from National Health Interview Survey, 2006–2014. A) Total population, B) ASCVD population

Total population								
	All-cause mortality				CVD mortality			
	*HR (95% CI)*	*HR (95% CI)*	*HR (95% CI)*	*HR (95% CI)*	*HR (95% CI)*	*HR (95% CI)*	*HR (95% CI)*	*HR (95% CI)*
Education	Model 1	Model 2	Model 3	Model 4	Model 1	Model 2	Model 3	Model 4
> College	Reference	Reference	Reference	Reference	Reference	Reference	Reference	Reference
Some College	1.18 (1.09, 1.27)	1.33 (1.24, 1.44)	1.16 (1.07, 1.25)	1.05 (0.97, 1.14)	1.32 (1.13, 1.54)	1.45 (1.26, 1.68)	1.17 (1.00, 1.38)	1.00 (0.84, 1.19)
High School or GED	1.84 (1.73, 1.96)	1.53 (1.44, 1.63)	1.22 (1.14, 1.30)	1.08 (1.00, 1.15)	1.89 (1.67, 2.14)	1.44 (1.26, 1.63)	1.11 (0.95, 1.30)	0.94 (0.80, 1.11)
< High School	2.91 (2.73, 3.11)	2.03 (1.89, 2.17)	1.39 (1.29, 1.50)	1.20 (1.11, 1.30)	3.54 (3.14, 4.00)	2.25 (1.97, 2.56)	1.51 (1.26, 1.81)	1.24 (1.04, 1.49)
ASCVD population								
	All-cause mortality				CVD mortality			
	*HR (95% CI)*	*HR (95% CI)*	*HR (95% CI)*	*HR (95% CI)*	*HR (95% CI)*	*HR (95% CI)*	*HR (95% CI)*	*HR (95% CI)*
Education	Model 1	Model 2	Model 3	Model 4	Model 1	Model 2	Model 3	Model 4
> College	Reference	Reference	Reference	Reference	Reference	Reference	Reference	Reference
Some College	1.15 (1.01, 1.29)	1.22 (1.08, 1.37)	1.09 (0.95, 1.26)	1.02 (0.89, 1.18)	1.21 (0.95, 1.53)	1.28 (1.00, 1.63)	1.07 (0.82, 1.41)	0.94 (0.71, 1.23)
High School or GED	1.37 (1.24, 1.51)	1.31 (1.19, 1.45)	1.13 (1.00, 1.28)	1.05 (0.93, 1.19)	1.30 (1.06, 1.60)	1.24 (1.01, 1.53)	1.02 (0.79, 1.31)	0.92 (0.71, 1.20)
< High School	1.76 (1.59, 1.96)	1.55 (1.39, 1.72)	1.21 (1.06, 1.38)	1.09 (0.95, 1.25)	2.03 (1.64, 2.53)	1.76 (1.40, 2.21)	1.38 (1.04, 1.82)	1.20 (0.90, 1.60)

Model 1: Unadjusted

Model 2: Adjusted for age, sex, and race/ethnicity

Model 3: Adjusted for Model 2 plus and income and insurance

Model 4: Adjusted for Model 3 and cardiovascular risk factors

*Abbreviations*: *CVD* cardiovascular disease, *CI* confidence interval, General Equivalency Diploma, *HR* hazard ratio, *HS* high school

The education-mortality association was relatively stronger in the total population than the ASCVD population, with relatively higher HR for < HS (vs. ≥ College) for all-cause and CVD mortality. However, the education-mortality association was not statistically significant for other educational attainment levels in both the total and ASCVD populations. The observed association was attenuated after adjusting for CVD risk factors (Model 4) in the total population and was not statistically significant in the ASCVD population for < HS (vs. ≥ College).

### Subgroup analyses

We found similar patterns of higher mortality risk with lower educational attainment across demographic groups (age, sex, and race/ethnicity) (Supplementary Tables S1-S2, Additional file 1). In the supplementary analyses, we consistently found higher mortality risk associated with lower educational attainment across age, sex and race/ethnicity strata. Consistent with prior literature, AAMRs were higher for males and NHB, compared to females and NHW/Hispanic, respectively. Overall, the observed association between educational attainment and mortality was relatively stronger in the non-elderly, females and NHB subgroups, compared to their counterparts.

## Discussion

To our knowledge, this is the largest nationally representative study evaluating the longitudinal impact (approximately 10 years) of educational attainment on all-cause and CVD mortality in the general adult population and adults with ASCVD in the US. We also reported the education-mortality association across key sociodemographic subgroups including age, sex, and race/ethnicity strata. We observed an inverse relationship between educational attainment and risk of all-cause and CVD in both the total and ASCVD populations. Education < HS was associated with up to 1.2 to 1.5-fold higher risk of all mortality types compared to ≥ College for both total and ASCVD populations when adjusted for age, sex, race/ethnicity, income, and insurance status. Similar patterns were seen across age, sex, and race/ethnicity strata. Education < HS was associated with up to 1.4 to 1.5-fold and 1.2 to 1.4-fold higher risk of all mortality types compared to ≥ College in the total population and ASCVD population, respectively, when adjusted for insurance and income (Model 3). Upon further adjustment for CVD risk factors (Model 4), the education-mortality associations were attenuated in the total population and not statistically significant in the ASCVD population for all-cause and CVD mortality.

### Education-mortality gradient: influential factors

Based on prior studies, lower educational attainment is associated with lower life expectancy and higher mortality [10–15], especially in those with ASCVD [7, 20]. However, the effect of educational attainment on mortality is multi-factorial. First, lower educational attainment is associated with lower health literacy [20–23]. Health literacy provides individuals a better understanding and utilization of public health and preventive CVD recommendations and has been associated with improved health behaviors. Better health literacy has been associated with health behaviors such as smoking cessation, healthy food consumption and greater adherence to dietary and physical activity guidelines, which lead to less health care demand and potentially better quality of life[20–23].Better health behaviors, especially reduction in smoking have been shown to improve all-cause and CVD mortality [20, 21]. Second, income and access to care are associated with educational attainment and mortality; those with higher income and educational attainment have better access to care and lower mortality rates [22]. Third, age and racial disparities may pose socioeconomic challenges in obtaining higher educational attainment and insurance and access to care (discussed below). There are other hypothesized mechanisms via which educational attainment may impact mortality that are poorly defined and require further investigation.

In our study, the education-mortality gradient was stronger in the total vs. the ASCVD population, with higher HRs at nearly each level of educational attainment in the former group when adjusting for age, sex, and race/ethnicity (Model 2). It is possible the education-mortality association was diminished in the presence of ASCVD as there is a higher risk at baseline independent of other factors, so that the influence of an upstream factor such as education is slightly diminished. After adjusting for insurance and income (Model 3), the gradient was more attenuated in the total population compared to the ASCVD population, suggesting educational attainment has a stronger impact on mortality in the ASCVD population vs. the total population. The education-mortality association was further diminished in the total population and not statistically significant in the ASCVD population after adjusting for CVD risk factors (Model 4). These results are expected as there may be a higher risk present at baseline independent of other factors in the ASCVD population vs the total population in addition to CVD risk factors being established clinical predictors of CVD [5, 6, 10]. Another factor that likely contributed to the confidence interval including the null value is the much smaller number of participants in this subgroup, which resulted in less statistical precision compared with the total population. Further investigation is needed to better understand the association of education attainment with mortality among individuals with established ASCVD, especially as it relates to preventive care (e.g., lifestyle interventions, medical therapies, access to healthcare) and overall comorbidity burden.

### Trends in demographic subgroups: age, sex, and race/ethnicity

The educational-mortality association was consistent across various sociodemographic subgroups. In the total population, there was a higher risk of all-cause mortality for ages < 40 vs. ≥ 40 after adjusting for SDOH and CVD risk factors (Supplementary Tables S1-S2, Models 3–4, Additional file 1). In the ASCVD population, many of these associations were not statistically significant after adjusting for SDOH and CVD risk factors (Supplementary Tables S1-S2, Models 3–4, Additional file 1).

Population level mortality risk assessments traditionally focus on established risk factors such as clinical indicators of physical and mental health without due attention to key “upstream” SDOH such as educational attainment. Age and associated mortality risk factors such as medical comorbidities, genetics, and cumulative psychosocial stress may alter the effects of educational attainment in late life [24–28]. Further, higher educational attainment is strongly predictive of stable employment, which may improve access to care such as via employer-sponsored health coverage; lower educational attainment and stable employment may restrict access to care and increase the risk of adverse health outcomes including mortality. These effects may be blunted after the age of 65 [29], given access to Medicare and other public benefit programs. Therefore, once accounting for SDOH and CVD risk factors, the education-mortality risk is blunted in ages < 40 in the total population. Unlike ages < 40, which had very few CVD events [30], ages > 65 were observed to have a statistically significant CVD mortality risk in the ASCVD population after adjusting for CVD risk factors, which is expected given multi-morbidity with advanced age and CVD risk factors being clinical predictors of CVD [31].

Regarding sex, the education-mortality association was similar for both men and women in the total population for all-cause and CVD mortality for < HS vs. ≥ College when adjusting for SDOH and CVD risk factors (Supplementary Tables S1-S2, Model 3–4, Additional file 1). However, the impact of educational attainment on all-cause and CVD mortality was relatively more robust in women vs. men in the ASCVD population after adjusting for SDOH and CVD risk factors (Supplementary Table S1-S2, Model 3–4, Additional file 1). Sex differences in education-mortality association may be explained by sex-related risk factors such as pregnancy or hormonal influences, which may affect CV physiology [32, 33] and variable health behaviors, screening practices, and presentations of ASCVD in men and women that may alter diagnosis and management, contributing to adverse CVD outcomes in women [34–37].”

Similarly, we found a higher mortality risk for non-Hispanic Black compared to other racial/ethnic groups (Supplementary Table S1-S2, Model 2, Additional file 1), consistent with prior studies [37–39]. However, after adjusting for SDOH (Model 3), this risk is comparable to NHW. These findings reflect the persistent social adversity faced by racial/ethnic minority populations, suggesting educational attainment may be driven by or contributes to a ‘cumulative’ social burden in explaining the adverse outcomes experienced by the non-Hispanic Black population. Some mechanisms for these disparities may include disproportionate impact of adverse SDOH, including neighborhood disadvantage, housing/overall economic instability, and structural racism such as redlining policies and other forms of racial/ethnic discrimination on socially vulnerable populations, which may restrict access to opportunities for educational attainment and potentially worsen the negative impact of low educational attainment on mortality via increased risk of established CV risk factors such as obesity, diabetes, hypertension and others [40, 41]. Additionally, racial differences between non-Hispanic Black and other races extend to CVD therapies, including treatment of hypertension, coronary artery disease, arrhythmias, and heart failure [42]. As seen in the primary analyses, adjustment for income and insurance in adjusted models attenuated the observed sociodemographic disparities in mortality considerably, further highlighting the importance of cumulative SDOH burden in explaining health disparities by individual socioeconomic predictors. Future studies should focus on further understanding the race-education-mortality association and elucidating the impact of structural inequities such as lack of access to education for racial/ethnic minority subgroups.

### Potential approaches to reduce the education-mortality gradient

The factors mentioned above merit further investigation as their effect on the education-mortality association may be unique. These cumulative results necessitate upstream interventions, which can help improve midstream determinants over time to improve educational attainment and reduce mortality rates. Upstream interventions include social reform and public health policies to 1) improve educational attainment by targeting key demographics and SDOH burden to improve CVD and mortality and 2) reduce high risk health behaviors (smoking, drinking, etc.). These interventions will ultimately heighten educational attainment as an important social determinant of mortality and inform evidence-based CVD risk stratification and prevention strategies. Improving access to education by supporting schools and colleges serving socioeconomically disadvantaged subgroups can reduce dropout rates and improve graduation rates, bridging the societal gap in educational attainment with subsequent economic and health outcomes. Therefore, it is important to have policies supporting socially vulnerable communities to facilitate access to education through direct/indirect financial support and incentivizing higher educational attainment with easy access to loans/repayment policies. An example of an upstream intervention includes the 2015 Every Student Succeeds Act, establishing accountability of schools towards creating equitable educational attainment in the United States. While the impact of this bill on educational attainment is yet to be seen, it highlights current policies that can potentially narrow the education-mortality gap [43]. Additionally, there are several programs that support historically black colleges and universities in improving student retention in underserved communities. Examples include the United Negro College Fund and Thurgood Marshall College Fund that provides financial assistance to students, academic/career support services, and institutional development resources [44].

These results prompt the following questions: 1) how can existing, predominantly clinical risk prediction algorithms be remodeled for improved mortality prediction using educational attainment?, 2) how does educational attainment interact with other SDOH such as income, healthcare/barriers to care, neighborhood environment, and community/social context such as social support mechanisms to impact mortality risk?, and 3) from a policy standpoint, are there evidence-based interventions aiding the US population in mitigating the education-mortality gap? Based on the existing AHA/ACC guidelines for preventative cardiology [45], educational attainment is not a targeted SDOH for CVD; however, we hope this study attracts greater attention towards the education-mortality relationship in the ASCVD population. Our results may inform future interventions targeting educational attainment in CVD prevention frameworks – both from a health literacy standpoint and via greater advocacy efforts for increased access to educational attainment to improve population cardiovascular health.

### Strengths

This study is the most recent, large-scale, longitudinal assessment of the association between educational attainment and all-cause and CVD mortality using a disaggregated 4-level educational attainment variable. Ours is the one of the few population-based, national studies to report the risk of both all-cause and CVD mortality associated with varying levels of educational attainment in the general US adult population, and in adults with ASCVD. Other major strengths include a large sample size with statistically robust estimates, longitudinal analysis of the educational attainment effect over a 10-year follow up period, a disaggregated 4-level educational attainment variable, and the analyses stratified by major sociodemographic factors (age, sex, and race/ethnicity) and other SDOH (insurance and income).

### Limitations

Our study is not without limitations. Other racial/ethnic subgroups (Asian, native Americans, etc.) were not included in the analysis given small available sample sizes and potentially underpowered and non-representative mortality analyses (especially for CVD mortality) for these population subgroups in the NHIS database. Additionally, while higher risk of mortality is associated with lower educational attainment with regional/geographic variation [46, 47], assessment of regional variation in the education-mortality gradient was outside the scope of our study. Given the self-reported nature of the data, there is potential for under-reporting and misclassification. The prevalence of low educational attainment is likely to be under-estimated, potentially resulting in a bias of our estimates towards the null. Despite these expected limitations of survey datasets, the NHIS remains a reliable source of information on the health of the non-institutionalized US population and has been used extensively in social epidemiology and health outcomes research previously [48, 49]. However, the NDI has been previously shown to overrepresent CVD mortality [50]. Despite this limitation, the NDI remains the most reliable source of information for mortality for large national studies such as the NHIS and National Health and Nutrition Examination Survey (NHANES). Lastly, these results do not provide causal mechanisms for the relationship between educational attainment and mortality but indicate pathways to be investigated in future studies.

## Conclusions

Lower educational attainment is strongly associated with all-cause and CVD mortality in the total and ASCVD populations in the US. Despite some variation, the impact of low educational attainment on mortality risk is seen consistently across sociodemographic strata. Our study suggests that enhanced upstream interventions to improve access to high quality education in the US may have a significant impact on life expectancy. Future efforts to understand persistent disparities in CVD and all-cause mortality should pay close attention to the role of education, and include educational attainment as an independent predictor in mortality risk prediction algorithms.

## Supplementary Information

**Additional file 1:** This supplementary file contains additional tables that relate to educational attainment and risk of mortality and age-adjusted mortality rates for the total and ASCVD populations. **S1.** Total Population: Education and Risk of Mortality, by age, sex, and race/ethnicity from the National Health Interview Survey, 2006-2014. **S2.** ASCVD Population: Education and Risk of Mortality, by age, sex, and race/ethnicity from the National Health Interview Survey, 2006-2014. **S3.** Age-adjusted Mortality Rate (per 100,000) for the Total and ASCVD populations by All-Cause and CVD Mortality.

## Data Availability

The datasets generated and analyzed during the current study are available in the National Health Institute (https://www.cdc.gov/nchs/nhis/data-questionnaires-documentation.htm).

## Abbreviations

AAMR: Age-adjusted mortality rates
ASCVD: Atherosclerotic cardiovascular disease
CVD: Cardiovascular disease
ICD: International Classification of Disease
NDI: National Death Index
NHIS: National Health Interview Survey
