# Supplementary material for: Low educational attainment is associated with higher all-cause and cardiovascular mortality in the United States adult population
Source: BMC Public Health. 2023 May 16;23:900. doi: 10.1186/s12889-023-15621-y (PMC10186682; doi:10.1186/s12889-023-15621-y)
Supplement: Supplementary file 1 — Additional file 1: This supplementary file contains additional tables that relate to educational attainment and risk of mortality and age-adjusted mortality rates for the total and ASCVD populations. S1. Total Population: Education and Risk of Mortality, by age, sex, and race/ethnicity from the National Health Interview Survey, 2006-2014. S2. ASCVD Population: Education and Risk of Mortality, by age, sex, and race/ethnicity from the National Health Interview Survey, 2006-2014. S3. Age-adjusted Mortality Rate (per 100,000) for the Total and ASCVD populations by All-Cause and CVD Mortality. [file 12889_2023_15621_MOESM1_ESM.docx]

S1. Total Population: Education and Risk of Mortality, by age, sex and race/ethnicity from the National Health Interview Survey, 2006-14.

|  |  | *All-Cause Mortality* | | | | *CVD Mortality* | | | |
| --- | --- | --- | --- | --- | --- | --- | --- | --- | --- |
|  |  | *HR (95% CI)* | *HR (95% CI)* | *HR (95% CI)* | *HR (95% CI)* | *HR (95% CI)* | *HR (95% CI)* | *HR (95% CI)* | *HR (95% CI)* |
|  | Education | Model 1* | Model 2** | Model 3*** | Model 4**** | Model 1* | Model 2** | Model 3*** | Model 4**** |
| 18-39 | > College | Reference | Reference | Reference | Reference | Reference | Reference | Reference | Reference |
|  | Some College | 1.16 (0.87, 1.54) | 1.33 (0.98, 1.79) | 1.19 (0.87, 1.62) | 1.11 (0.80, 1.55) | 0.77 (0.26, 2.27) | 1.27 (0.35, 4.56) | 0.74 (0.16, 3.47) | 0.53 (0.10, 2.74) |
|  | High School or GED | 2.10 (1.62, 2.73) | 2.13 (1.63, 2.79) | 1.55 (1.15, 2.07) | 1.39 (1.02, 1.90) | 1.52 (0.55, 4.14) | 1.84 (0.58, 5.85) | 0.90 (0.20, 4.04) | 0.73 (0.16, 3.34) |
|  | < High School | 3.36 (2.59, 4.35) | 3.07 (2.25, 4.20) | 1.97 (1.39, 2.78) | 1.79 (1.27, 2.54) | 3.48 (1.48, 8.20) | 3.38 (1.13, 10.16) | 1.20 (0.28, 5.20) | 0.98 (0.23, 4.15) |
|  | Education | Model 1* | Model 2** | Model 3*** | Model 4**** | Model 1* | Model 2** | Model 3*** | Model 4**** |
| 40-65 | > College | Reference | Reference | Reference | Reference | Reference | Reference | Reference | Reference |
|  | Some College | 1.48 (1.30, 1.68) | 1.46 (1.29, 1.67) | 1.14 (0.99, 1.31) | 0.99 (0.86, 1.14) | 1.65 (1.23, 2.22) | 1.59 (1.17, 2.17) | 1.13 (0.79, 1.60) | 0.97 (0.68, 1.39) |
|  | High School or GED | 1.93 (1.72, 2.17) | 1.91 (1.69, 2.15) | 1.34 (1.18, 1.53) | 1.11 (0.98, 1.26) | 1.79 (1.39, 2.32) | 1.84 (1.40, 2.42) | 1.24 (0.90, 1.71) | 0.98 (0.72, 1.35) |
|  | < High School | 2.70 (2.37, 3.07) | 2.68 (2.32, 3.09) | 1.33 (1.15, 1.56) | 1.07 (0.91, 1.24) | 2.53 (1.92, 3.32) | 2.58 (1.93, 3.46) | 1.11 (0.76, 1.63) | 0.84 (0.57, 1.22) |
|  | Education | Model 1* | Model 2** | Model 3*** | Model 4**** | Model 1* | Model 2** | Model 3*** | Model 4**** |
| 65+ | > College | Reference | Reference | Reference | Reference | Reference | Reference | Reference | Reference |
|  | Some College | 1.23 (1.12, 1.35) | 1.19 (1.08, 1.31) | 1.13 (1.03, 1.25) | 1.06 (0.96, 1.16) | 1.43 (1.21, 1.69) | 1.35 (1.14, 1.60) | 1.18 (0.96, 1.44) | 1.01 (0.82, 1.24) |
|  | High School or GED | 1.30 (1.20, 1.41) | 1.22 (1.12, 1.32) | 1.10 (1.01, 1.20) | 1.02 (0.93, 1.11) | 1.39 (1.20, 1.61) | 1.23 (1.06, 1.44) | 1.03 (0.85, 1.25) | 0.91 (0.74, 1.11) |
|  | < High School | 1.83 (1.70, 1.98) | 1.59 (1.46, 1.73) | 1.35 (1.22, 1.48) | 1.21 (1.09, 1.33) | 2.42 (2.10, 2.79) | 2.02 (1.73, 2.35) | 1.63 (1.33, 2.01) | 1.40 (1.13, 1.73) |
|  | Education | Model 1* | Model 2** | Model 3*** | Model 4**** | Model 1* | Model 2** | Model 3*** | Model 4**** |
| Male | > College | Reference | Reference | Reference | Reference | Reference | Reference | Reference | Reference |
|  | Some College | 1.13 (1.03, 1.25) | 1.40 (1.27, 1.54) | 1.24 (1.12, 1.37) | 1.12 (1.00, 1.24) | 1.30 (1.07, 1.57) | 1.58 (1.31, 1.92) | 1.20 (0.96, 1.49) | 0.96 (0.76, 1.22) |
|  | High School or GED | 1.54 (1.42, 1.68) | 1.57 (1.44, 1.70) | 1.25 (1.14, 1.37) | 1.09 (0.99, 1.19) | 1.45 (1.22, 1.71) | 1.43 (1.20, 1.70) | 1.03 (0.83, 1.28) | 0.86 (0.68, 1.08) |
|  | < High School | 2.46 (2.25, 2.68) | 2.03 (1.86, 2.22) | 1.41 (1.28, 1.56) | 1.22 (1.10, 1.35) | 2.76 (2.33, 3.27) | 2.24 (1.87, 2.69) | 1.48 (1.16, 1.88) | 1.21 (0.95, 1.54) |
|  | Education | Model 1* | Model 2** | Model 3*** | Model 4**** | Model 1* | Model 2** | Model 3*** | Model 4**** |
| Female | > College | Reference | Reference | Reference | Reference | Reference | Reference | Reference | Reference |
|  | Some College | 1.26 (1.12, 1.42) | 1.25 (1.12, 1.41) | 1.06 (0.95, 1.19) | 0.99 (0.88, 1.11) | 1.39 (1.09, 1.77) | 1.30 (1.02, 1.64) | 1.15 (0.87, 1.52) | 1.06 (0.79, 1.41) |
|  | High School or GED | 2.25 (2.04, 2.48) | 1.48 (1.33, 1.63) | 1.17 (1.06, 1.30) | 1.06 (0.95, 1.17) | 2.61 (2.17, 3.15) | 1.41 (1.17, 1.71) | 1.20 (0.94, 1.54) | 1.05 (0.82, 1.36) |
|  | < High School | 3.54 (3.22, 3.89) | 2.00 (1.80, 2.22) | 1.37 (1.22, 1.53) | 1.18 (1.06, 1.32) | 4.82 (4.04, 5.75) | 2.20 (1.82, 2.65) | 1.54 (1.19, 1.99) | 1.29 (1.00, 1.67) |
|  | Education | Model 1* | Model 2** | Model 3*** | Model 4**** | Model 1* | Model 2** | Model 3*** | Model 4**** |
| NHW | > College | Reference | Reference | Reference | Reference | Reference | Reference | Reference | Reference |
|  | Some College | 1.26 (1.15, 1.38) | 1.32 (1.21, 1.44) | 1.15 (1.05, 1.26) | 1.04 (0.95, 1.14) | 1.37 (1.15, 1.63) | 1.42 (1.21, 1.67) | 1.16 (0.96, 1.40) | 0.97 (0.80, 1.19) |
|  | High School or GED | 2.01 (1.86, 2.16) | 1.51 (1.41, 1.63) | 1.21 (1.12, 1.31) | 1.06 (0.98, 1.15) | 2.10 (1.81, 2.43) | 1.45 (1.25, 1.68) | 1.11 (0.93, 1.34) | 0.93 (0.77, 1.13) |
|  | < High School | 3.81 (3.52, 4.12) | 2.08 (1.92, 2.26) | 1.42 (1.29, 1.56) | 1.20 (1.09, 1.32) | 4.75 (4.08, 5.53) | 2.32 (2.00, 2.69) | 1.55 (1.25, 1.92) | 1.25 (1.01, 1.55) |
|  | Education | Model 1* | Model 2** | Model 3*** | Model 4**** | Model 1* | Model 2** | Model 3*** | Model 4**** |
| NHB | > College | Reference | Reference | Reference | Reference | Reference | Reference | Reference | Reference |
|  | Some College | 1.18 (0.97, 1.44) | 1.45 (1.20, 1.75) | 1.20 (0.99, 1.46) | 1.13 (0.92, 1.39) | 1.36 (0.89, 2.07) | 1.64 (1.08, 2.49) | 1.56 (0.97, 2.51) | 1.42 (0.88, 2.29) |
|  | High School or GED | 1.79 (1.48, 2.16) | 1.58 (1.32, 1.90) | 1.21 (1.00, 1.46) | 1.12 (0.92, 1.36) | 1.64 (1.14, 2.37) | 1.39 (0.96, 2.00) | 1.20 (0.79, 1.83) | 1.09 (0.70, 1.70) |
|  | < High School | 4.03 (3.38, 4.81) | 2.13 (1.79, 2.55) | 1.39 (1.16, 1.68) | 1.31 (1.08, 1.58) | 4.96 (3.46, 7.10) | 2.26 (1.57, 3.25) | 1.61 (1.03, 2.50) | 1.46 (0.94, 2.27) |
|  | Education | Model 1* | Model 2** | Model 3*** | Model 4**** | Model 1* | Model 2** | Model 3*** | Model 4**** |
| Hispanic | > College | Reference | Reference | Reference | Reference | Reference | Reference | Reference | Reference |
|  | Some College | 0.75 (0.57, 0.99) | 1.00 (0.77, 1.31) | 0.95 (0.73, 1.25) | 0.93 (0.70, 1.23) | 0.91 (0.50, 1.64) | 1.32 (0.73, 2.36) | 0.63 (0.32, 1.28) | 0.59 (0.30, 1.17) |
|  | High School or GED | 1.15 (0.90, 1.48) | 1.25 (0.97, 1.61) | 1.13 (0.88, 1.45) | 1.06 (0.82, 1.36) | 0.97 (0.58, 1.63) | 1.01 (0.60, 1.68) | 0.86 (0.49, 1.52) | 0.79 (0.45, 1.40) |
|  | < High School | 1.76 (1.43, 2.16) | 1.46 (1.20, 1.76) | 1.24 (1.01, 1.52) | 1.15 (0.93, 1.41) | 2.21 (1.48, 3.31) | 1.58 (1.05, 2.38) | 1.19 (0.76, 1.85) | 1.03 (0.65, 1.62) |

Abbreviations: HR, Hazard Ratios; CI, confidence interval

| * Unadjusted | |
| --- | --- |
| ** Adjusted for age, sex and race/ethnicity | |
| *** Adjusted for Model 2 + insurance and income |  |
| **** Adjusted for Model 3 + Cardiovascular risk factors |  |

| S2. ASCVD Population: Education and Risk of Mortality, by age, sex and race/ethnicity from the National Health Interview Survey, 2006-14. | | | | | | | | | |
| --- | --- | --- | --- | --- | --- | --- | --- | --- | --- |
|  |  | All-Cause Mortality | | | | CVD Mortality | | | |
|  |  | *HR (95% CI)* | *HR (95% CI)* | *HR (95% CI)* | *HR (95% CI)* | *HR (95% CI)* | *HR (95% CI)* | *HR (95% CI)* | *HR (95% CI)* |
|  | Education | Model 1* | Model 2** | Model 3*** | Model 4**** | Model 1* | Model 2** | Model 3*** | Model 4**** |
| 18-39 | > College | Reference | Reference | Reference | Reference | Reference | Reference | Reference | Reference |
|  | Some College | 1.20 (0.23, 6.34) | 1.00 (0.16, 6.41) | 0.89 (0.15, 5.45) | 0.94 (0.15, 5.69) | — | — | — | — |
|  | High School or GED | 1.93 (0.46, 8.15) | 1.90 (0.48, 7.49) | 1.27 (0.25, 6.51) | 1.24 (0.27, 5.73) | — | — | — | — |
|  | < High School | 3.47 (0.83, 14.48) | 3.44 (0.74, 15.93) | 2.23 (0.40, 12.47) | 1.91 (0.37, 9.96) | — | — | — | — |
|  | Education | Model 1* | Model 2** | Model 3*** | Model 4**** | Model 1* | Model 2** | Model 3*** | Model 4**** |
| 40-65 | > College | Reference | Reference | Reference | Reference | Reference | Reference | Reference | Reference |
|  | Some College | 1.24 (0.96, 1.59) | 1.27 (0.98, 1.67) | 1.04 (0.77, 1.39) | 0.89 (0.66, 1.20) | 1.19 (0.69, 2.06) | 1.04 (0.61, 1.80) | 0.92 (0.51, 1.66) | 0.83 (0.45, 1.53) |
|  | High School or GED | 1.73 (1.38, 2.19) | 1.88 (1.48, 2.40) | 1.32 (1.00, 1.75) | 1.12 (0.84, 1.48) | 1.27 (0.83, 1.96) | 1.36 (0.87, 2.13) | 1.13 (0.66, 1.91) | 1.01 (0.59, 1.72) |
|  | < High School | 2.03 (1.57, 2.62) | 2.23 (1.71, 2.91) | 1.19 (0.88, 1.59) | 1.04 (0.78, 1.40) | 1.48 (0.91, 2.40) | 1.60 (0.98, 2.60) | 0.93 (0.52, 1.68) | 0.83 (0.46, 1.52) |
|  | Education | Model 1* | Model 2** | Model 3*** | Model 4**** | Model 1* | Model 2** | Model 3*** | Model 4**** |
| 65+ | > College | Reference | Reference | Reference | Reference | Reference | Reference | Reference | Reference |
|  | Some College | 1.18 (1.03, 1.36) | 1.19 (1.04, 1.37) | 1.10 (0.94, 1.29) | 1.06 (0.90, 1.24) | 1.32 (1.00, 1.73) | 1.34 (1.01, 1.77) | 1.09 (0.79, 1.49) | 0.94 (0.68, 1.28) |
|  | High School or GED | 1.21 (1.08, 1.35) | 1.18 (1.05, 1.32) | 1.06 (0.93, 1.22) | 1.02 (0.89, 1.17) | 1.25 (0.97, 1.60) | 1.19 (0.92, 1.53) | 0.95 (0.70, 1.28) | 0.87 (0.64, 1.18) |
|  | < High School | 1.45 (1.30, 1.63) | 1.38 (1.22, 1.56) | 1.20 (1.03, 1.41) | 1.10 (0.93, 1.28) | 1.88 (1.48, 2.41) | 1.80 (1.39, 2.32) | 1.48 (1.09, 2.03) | 1.29 (0.94, 1.77) |
|  | Education | Model 1* | Model 2** | Model 3*** | Model 4**** | Model 1* | Model 2** | Model 3*** | Model 4**** |
| Male | > College | Reference | Reference | Reference | Reference | Reference | Reference | Reference | Reference |
|  | Some College | 1.21 (1.04, 1.41) | 1.25 (1.08, 1.46) | 1.11 (0.93, 1.33) | 1.02 (0.85, 1.23) | 1.23 (0.90, 1.67) | 1.22 (0.89, 1.67) | 0.93 (0.65, 1.32) | 0.75 (0.52, 1.09) |
|  | High School or GED | 1.30 (1.14, 1.48) | 1.33 (1.16, 1.52) | 1.10 (0.94, 1.28) | 1.00 (0.85, 1.17) | 1.18 (0.90, 1.56) | 1.21 (0.92, 1.61) | 0.87 (0.61, 1.23) | 0.76 (0.53, 1.09) |
|  | < High School | 1.66 (1.44, 1.91) | 1.48 (1.29, 1.71) | 1.12 (0.95, 1.34) | 1.00 (0.84, 1.19) | 1.85 (1.40, 2.45) | 1.71 (1.27, 2.31) | 1.22 (0.84, 1.76) | 1.04 (0.72, 1.51) |
|  | Education | Model 1* | Model 2** | Model 3*** | Model 4**** | Model 1* | Model 2** | Model 3*** | Model 4**** |
| Female | > College | Reference | Reference | Reference | Reference | Reference | Reference | Reference | Reference |
|  | Some College | 1.09 (0.90, 1.32) | 1.16 (0.96, 1.41) | 1.09 (0.87, 1.35) | 1.06 (0.84, 1.33) | 1.26 (0.86, 1.83) | 1.44 (0.99, 2.10) | 1.46 (0.94, 2.25) | 1.41 (0.90, 2.21) |
|  | High School or GED | 1.52 (1.29, 1.77) | 1.30 (1.12, 1.52) | 1.18 (0.98, 1.43) | 1.16 (0.96, 1.40) | 1.58 (1.18, 2.13) | 1.32 (0.98, 1.79) | 1.36 (0.93, 1.99) | 1.33 (0.90, 1.95) |
|  | < High School | 1.96 (1.67, 2.30) | 1.62 (1.38, 1.91) | 1.34 (1.08, 1.65) | 1.24 (1.00, 1.54) | 2.48 (1.83, 3.36) | 1.87 (1.36, 2.57) | 1.75 (1.15, 2.66) | 1.64 (1.08, 2.50) |
|  | Education | Model 1* | Model 2** | Model 3*** | Model 4**** | Model 1* | Model 2** | Model 3*** | Model 4**** |
| NHW | > College | Reference | Reference | Reference | Reference | Reference | Reference | Reference | Reference |
|  | Some College | 1.17 (1.02, 1.34) | 1.19 (1.05, 1.36) | 1.09 (0.93, 1.27) | 1.02 (0.87, 1.19) | 1.13 (0.86, 1.48) | 1.20 (0.91, 1.56) | 0.99 (0.73, 1.35) | 0.86 (0.63, 1.18) |
|  | High School or GED | 1.41 (1.26, 1.57) | 1.31 (1.18, 1.46) | 1.12 (0.98, 1.29) | 1.05 (0.92, 1.20) | 1.34 (1.06, 1.69) | 1.26 (1.00, 1.58) | 1.00 (0.76, 1.33) | 0.92 (0.69, 1.22) |
|  | < High School | 1.96 (1.74, 2.22) | 1.61 (1.43, 1.81) | 1.27 (1.10, 1.47) | 1.13 (0.97, 1.32) | 2.32 (1.82, 2.95) | 1.87 (1.47, 2.39) | 1.42 (1.05, 1.92) | 1.22 (0.89, 1.67) |
|  | Education | Model 1* | Model 2** | Model 3*** | Model 4**** | Model 1* | Model 2** | Model 3*** | Model 4**** |
| NHB | > College | Reference | Reference | Reference | Reference | Reference | Reference | Reference | Reference |
|  | Some College | 1.44 (1.01, 2.05) | 1.50 (1.05, 2.13) | 1.34 (0.89, 2.02) | 1.28 (0.84, 1.95) | 1.93 (0.89, 4.16) | 2.00 (0.92, 4.35) | 2.41 (1.01, 5.77) | 2.21 (0.93, 5.25) |
|  | High School or GED | 1.60 (1.14, 2.23) | 1.49 (1.07, 2.07) | 1.24 (0.82, 1.86) | 1.17 (0.77, 1.76) | 1.49 (0.77, 2.87) | 1.36 (0.72, 2.59) | 1.42 (0.65, 3.08) | 1.32 (0.60, 2.91) |
|  | < High School | 2.52 (1.82, 3.50) | 1.75 (1.26, 2.41) | 1.28 (0.84, 1.95) | 1.23 (0.80, 1.89) | 3.46 (1.79, 6.69) | 2.23 (1.12, 4.46) | 2.11 (0.91, 4.92) | 1.99 (0.86, 4.61) |
|  | Education | Model 1* | Model 2** | Model 3*** | Model 4**** | Model 1* | Model 2** | Model 3*** | Model 4**** |
| Hispanic | > College | Reference | Reference | Reference | Reference | Reference | Reference | Reference | Reference |
|  | Some College | 0.91 (0.57, 1.45) | 1.05 (0.68, 1.62) | 0.82 (0.49, 1.40) | 0.73 (0.41, 1.29) | 1.47 (0.61, 3.55) | 1.73 (0.72, 4.13) | 1.17 (0.39, 3.52) | 0.98 (0.32, 3.02) |
|  | High School or GED | 0.92 (0.60, 1.40) | 1.02 (0.66, 1.55) | 1.07 (0.63, 1.81) | 1.00 (0.60, 1.68) | 0.83 (0.34, 2.03) | 0.90 (0.36, 2.26) | 1.14 (0.38, 3.41) | 1.01 (0.35, 2.92) |
|  | < High School | 1.04 (0.73, 1.48) | 0.86 (0.61, 1.22) | 0.73 (0.48, 1.13) | 0.66 (0.43, 1.02) | 1.03 (0.49, 2.17) | 0.76 (0.36, 1.57) | 0.98 (0.46, 2.11) | 0.87 (0.40, 1.89) |

Abbreviations: HR, Hazard Ratios; CI, confidence interval

| * Unadjusted | |
| --- | --- |
| ** Adjusted for age, sex and race/ethnicity | |
| *** Adjusted for Model 2 + insurance and income |  |
| **** Adjusted for Model 3 + Cardiovascular risk factors |  |

| S3. Age-adjusted Mortality Rate (per 100,000) for the Total and ASCVD Populations by All-Cause and CVD Mortality. | | | | | | | | |
| --- | --- | --- | --- | --- | --- | --- | --- | --- |
| All-Cause Mortality | | | | | | | | |
|  | *Total Population* | | | | *ASCVD Population* | | | |
|  | < High School | High School or GED | Some College | > College | < High School | High School or GED | Some College | > College |
| Mortality Rates | 400.6 ( 386.7, 414.4) | 303.0 ( 292.1, 313.9) | 276.1 ( 262.4, 289.8) | 208.6 ( 200.3, 216.9) | 1446.7 ( 1362.8, 1530.6) | 1265.5 ( 1190.4, 1340.6) | 1177.1 ( 1078.8, 1275.3) | 984.0 ( 908.8, 1059.2) |
|  |  |  |  |  |  |  |  |  |
| Sub-group Analysis |  |  |  |  |  |  |  |  |
|  |  |  |  |  |  |  |  |  |
| Sex |  |  |  |  |  |  |  |  |
| *Male* | 490.7 ( 471.6, 509.9) | 375.9 ( 360.8, 391.0) | 340.9 ( 322.9, 358.9) | 250.7 ( 240.1, 261.2) | 1599.3 ( 1487.9, 1710.7) | 1400.5 ( 1304.7, 1496.2) | 1288.9 ( 1172.2, 1405.7) | 1060.4 ( 977.0, 1143.7) |
| *Female* | 333.8 ( 320.5, 347.2) | 255.7 ( 245.3, 266.1) | 231.9 ( 219.4, 244.4) | 170.5 ( 162.6, 178.4) | 1310.1 ( 1226.6, 1393.5) | 1147.2 ( 1069.1, 1225.3) | 1055.8 ( 960.9, 1150.7) | 868.6 ( 791.3, 945.9) |
| Race/Ethnicity |  |  |  |  |  |  |  |  |
| *Non-Hispanic White* | 411.7 ( 393.7, 429.6) | 306.7 ( 295.0, 318.4) | 279.4 ( 265.1, 293.8) | 211.5 ( 202.7, 220.3) | 1529.5 ( 1427.9, 1631.1) | 1289.4 ( 1210.6, 1368.1) | 1198.6 ( 1095.9, 1301.4) | 999.5 ( 921.4, 1077.6) |
| *Non-Hispanic Black* | 445.9 ( 421.1, 470.6) | 332.2 ( 310.4, 353.9) | 302.7 ( 279.7, 325.6) | 229.0 ( 213.0, 245.0) | 1493.0 ( 1336.8, 1649.2) | 1258.6 ( 1109.7, 1407.5) | 1170.0 ( 1014.6, 1325.5) | 975.7 ( 849.7, 1101.7) |
| *Hispanic* | 375.3 ( 348.5, 402.1) | 279.6 ( 257.4, 301.7) | 254.8 ( 231.7, 277.8) | 192.8 ( 175.9, 209.7) | 1146.0 ( 990.6, 1301.4) | 966.1 ( 820.5, 1111.7) | 898.1 ( 747.7, 1048.4) | 748.9 ( 629.5, 868.3) |
|  |  |  |  |  |  |  |  |  |
| CVD Morality | | | | | | | | |
|  | *Total Population* | | | | *ASCVD Population* | | | |
|  | < High School | High School or GED | Some College | > College | < High School | High School or GED | Some College | > College |
| Mortality Rates | 82.1 ( 75.8, 88.5) | 53.0 ( 48.4, 57.6) | 54.7 ( 48.8, 60.7) | 38.7 ( 35.4, 42.1) | 456.4 ( 399.3, 513.6) | 338.0 ( 295.7, 380.3) | 360.4 ( 303.7, 417.1) | 279.5 ( 233.2, 325.8) |
|  |  |  |  |  |  |  |  |  |
| Sub-group Analysis |  |  |  |  |  |  |  |  |
|  |  |  |  |  |  |  |  |  |
| Sex |  |  |  |  |  |  |  |  |
| *Male* | 110.5 ( 100.8, 120.2) | 72.4 ( 65.4, 79.5) | 74.1 ( 65.6, 82.6) | 50.2 ( 45.5, 54.9) | 533.8 ( 457.0, 610.5) | 394.5 ( 338.9, 450.2) | 414.1 ( 342.6, 485.6) | 313.9 ( 258.6, 369.2) |
| *Female* | 63.5 ( 57.6, 69.3) | 41.6 ( 37.5, 45.7) | 42.6 ( 37.5, 47.6) | 28.8 ( 25.9, 31.8) | 392.0 ( 335.9, 448.1) | 289.7 ( 247.4, 332.1) | 304.1 ( 251.4, 356.8) | 230.5 ( 188.3, 272.8) |
| Race/Ethnicity |  |  |  |  |  |  |  |  |
| *Non-Hispanic White* | 85.9 ( 77.5, 94.3) | 54.0 ( 49.0, 59.0) | 54.8 ( 48.8, 60.7) | 39.3 ( 35.7, 42.8) | 488.5 ( 416.7, 560.2) | 342.5 ( 298.4, 386.7) | 357.6 ( 300.9, 414.4) | 285.1 ( 235.7, 334.5) |
| *Non-Hispanic Black* | 94.0 ( 82.0, 106.1) | 59.1 ( 50.4, 67.7) | 59.9 ( 49.7, 70.1) | 43.0 ( 36.2, 49.7) | 468.2 ( 364.2, 572.3) | 328.4 ( 245.9, 410.8) | 342.8 ( 253.6, 432.0) | 273.3 ( 202.5, 344.1) |
| *Hispanic* | 72.1 ( 62.4, 81.8) | 45.3 ( 38.1, 52.5) | 46.0 ( 37.5, 54.4) | 33.0 ( 27.5, 38.4) | 355.6 ( 263.0, 448.2) | 249.4 ( 172.0, 326.8) | 260.4 ( 173.5, 347.2) | 207.6 ( 141.9, 273.3) |
